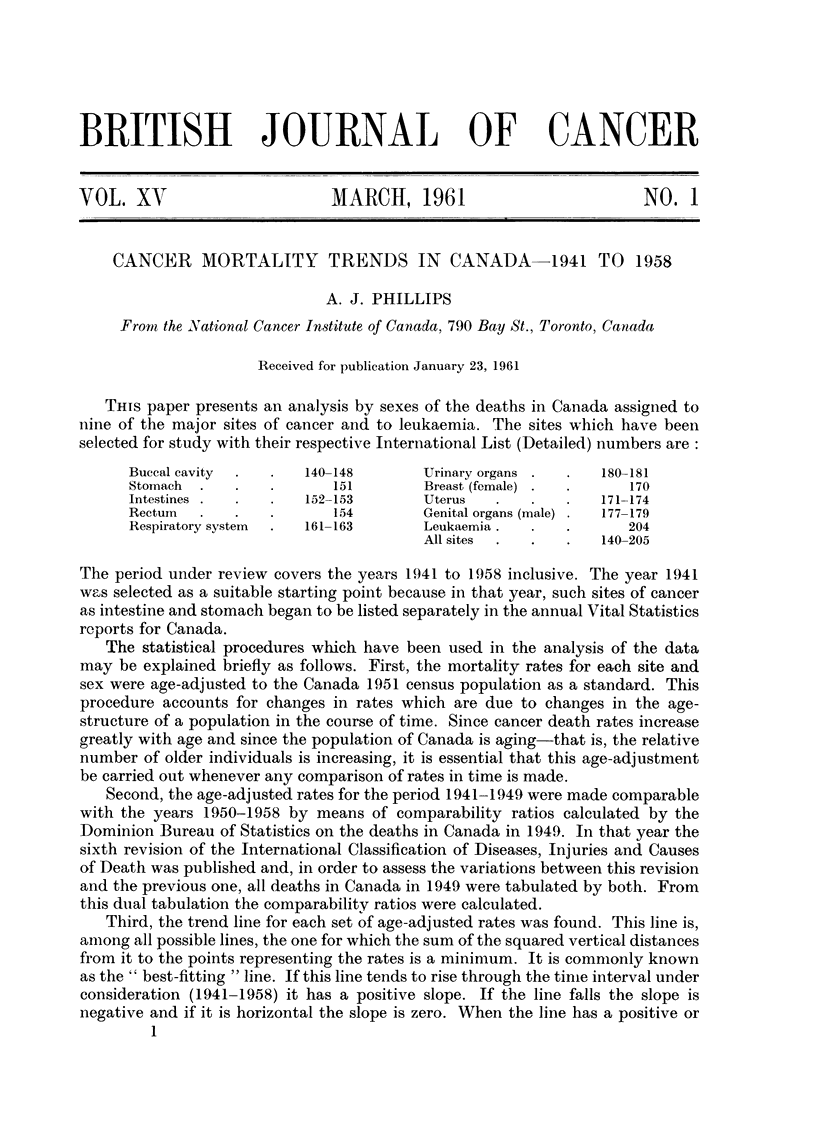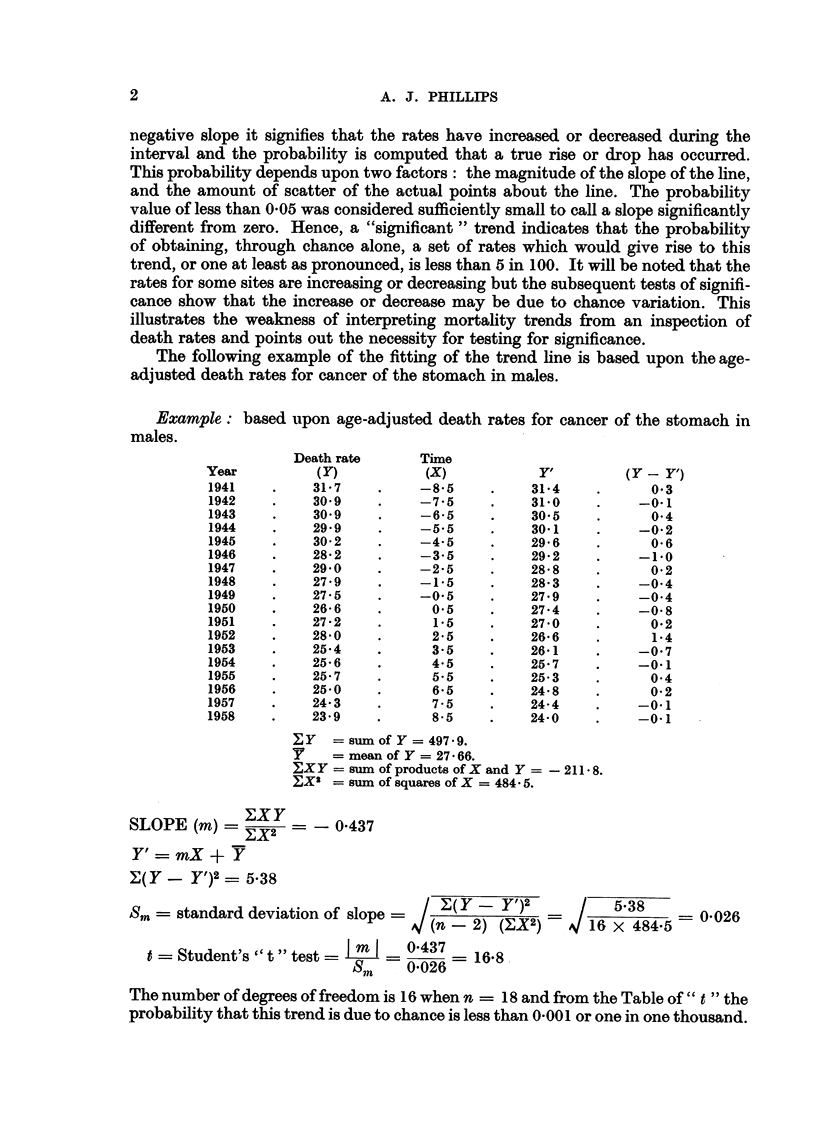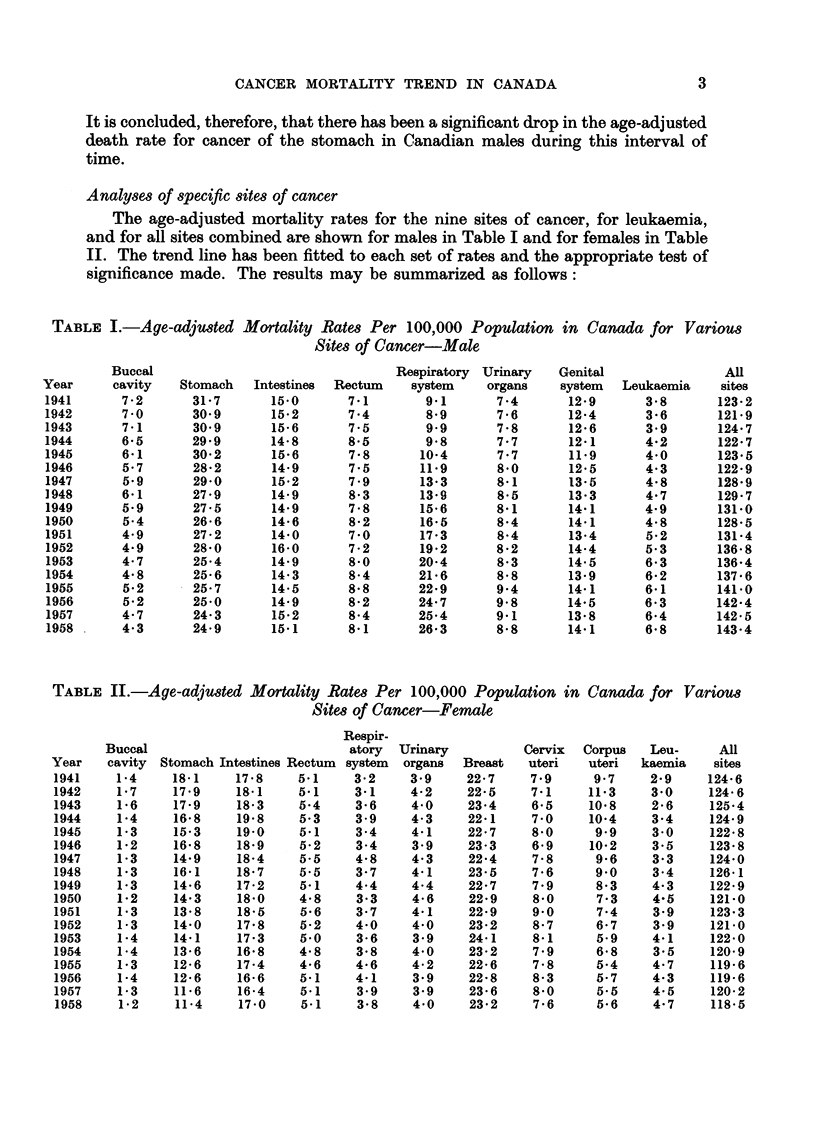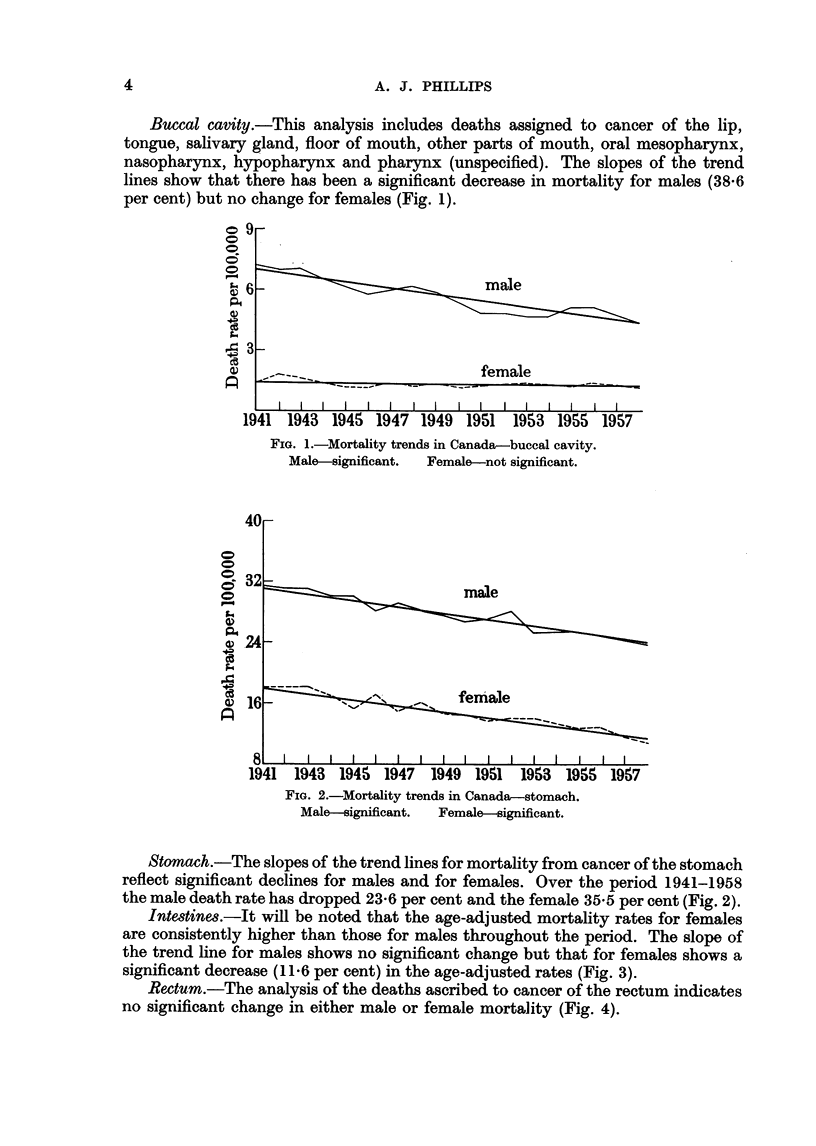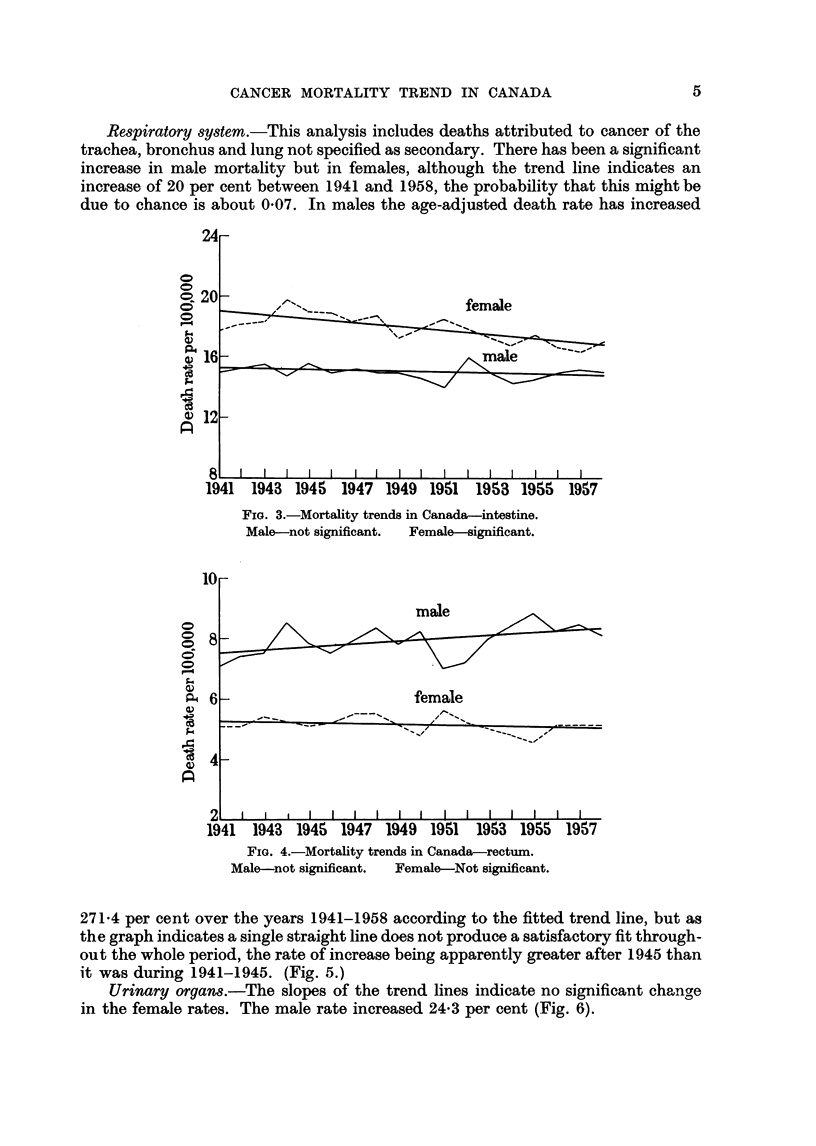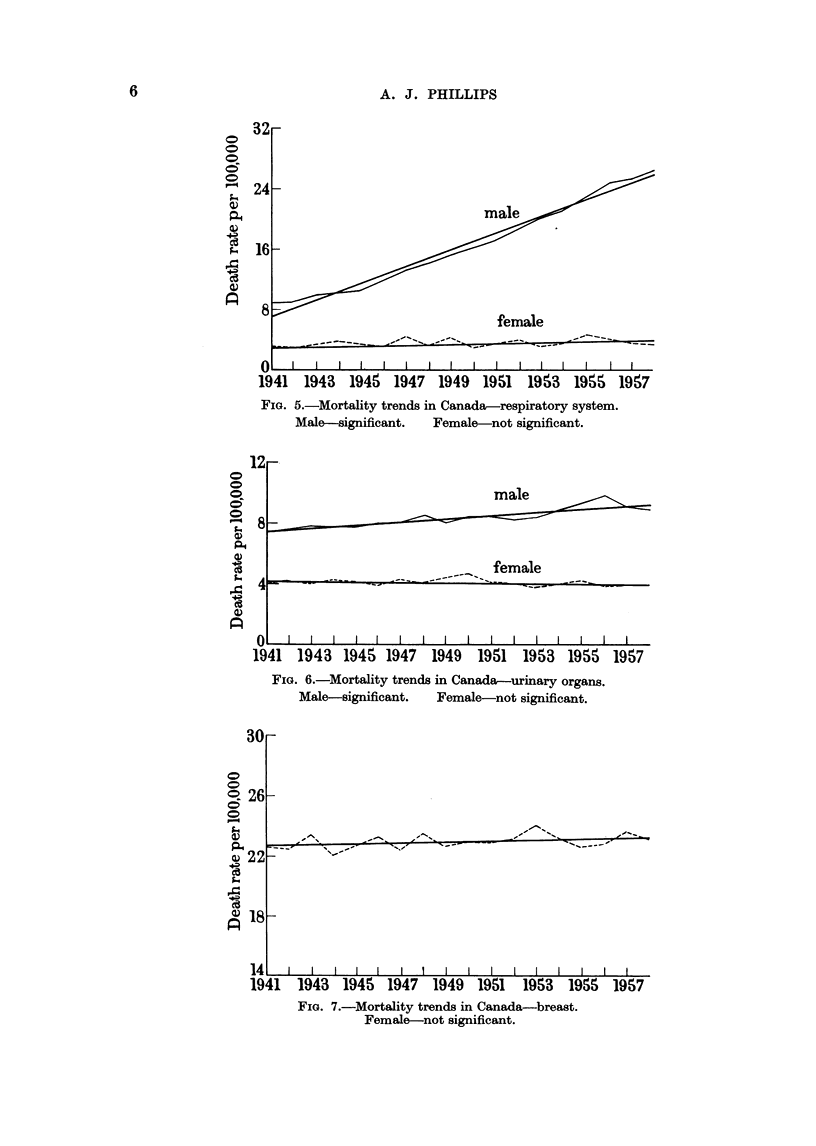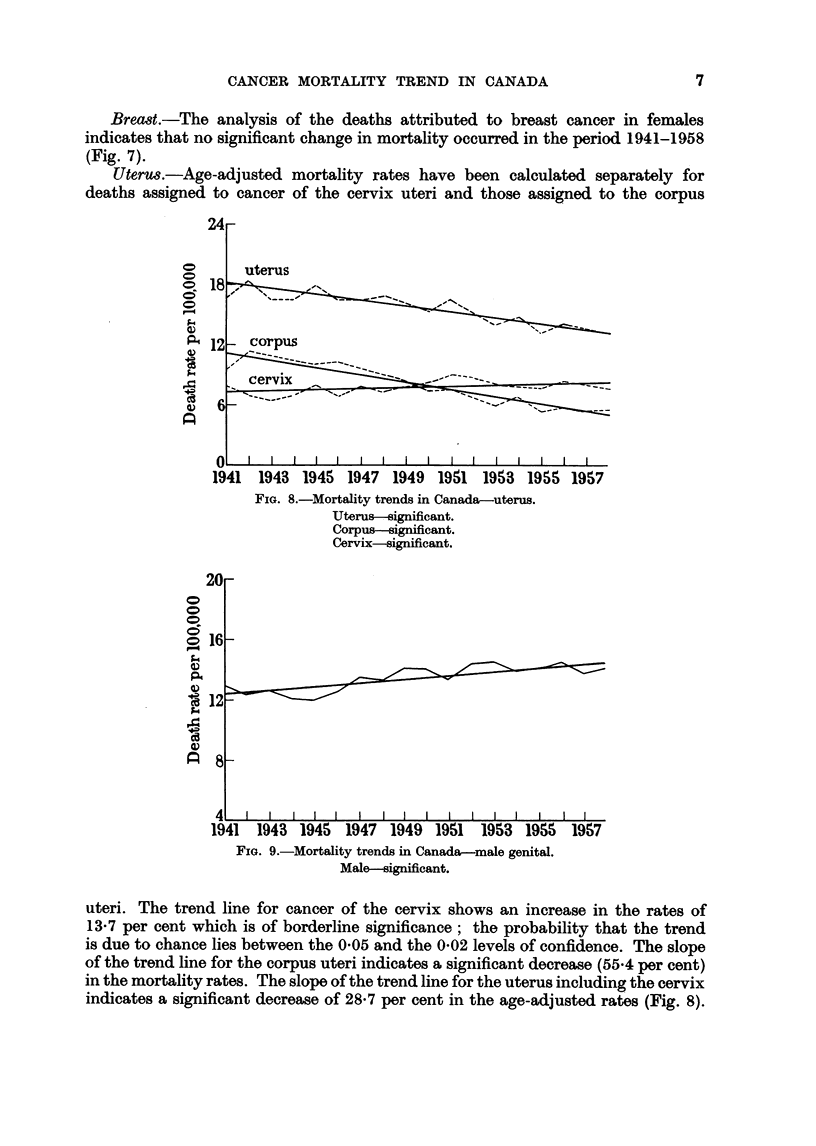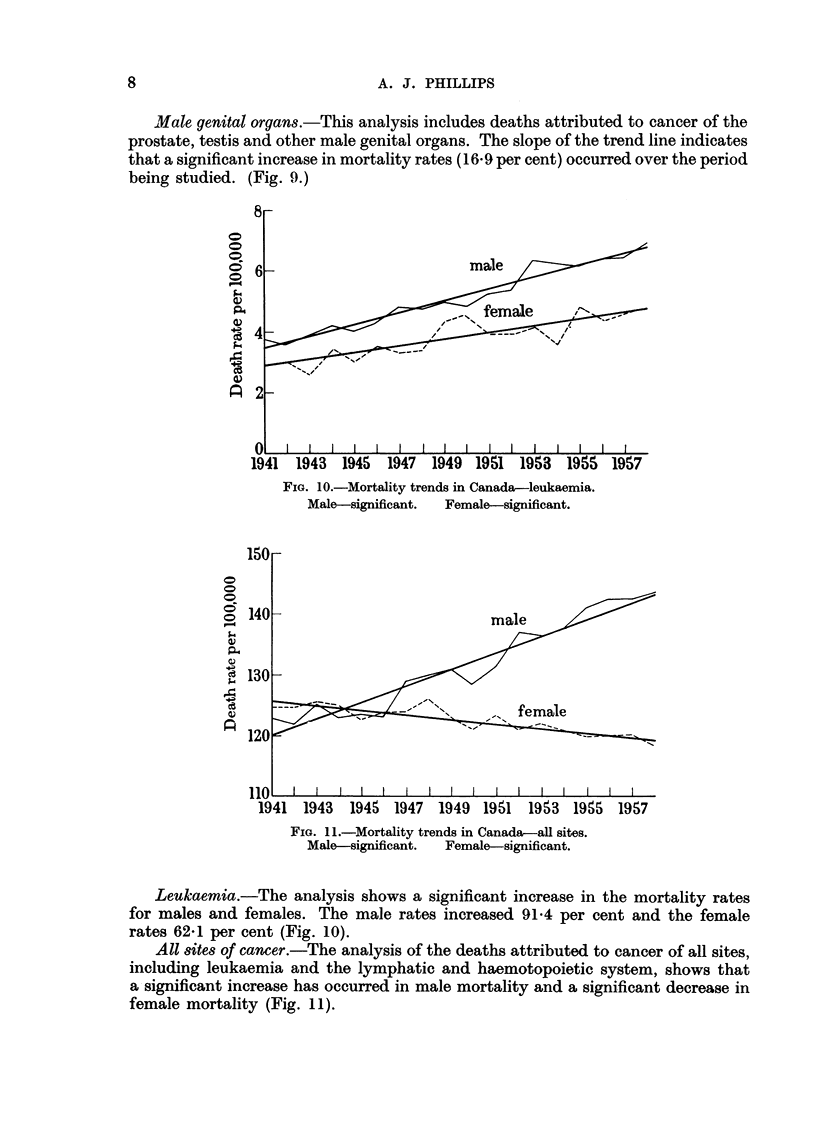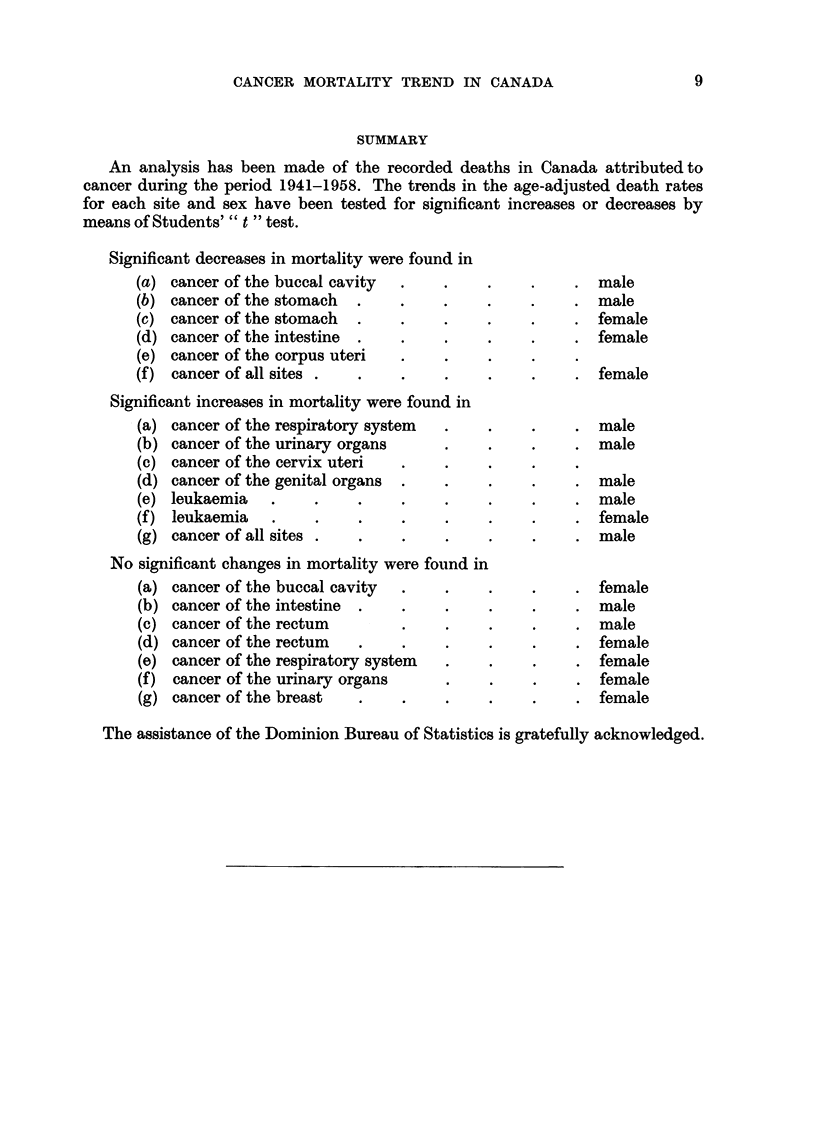# Cancer Mortality Trends in Canada—1941 to 1958

**DOI:** 10.1038/bjc.1961.1

**Published:** 1961-03

**Authors:** A. J. Phillips


BRITISH JOURNAL OF CANCER

VOL. XV MARCH, 1961 NO. 1

### CANCER MORTALITY TRENDS IN CANADA—1941 TO 1958

A. J. PHILLIPS

*From the National Cancer Institute of Canada, 790 Bay St., Toronto, Canada*

Received for publication January 23, 1961

THIS paper presents an analysis by sexes of the deaths in Canada assigned to nine of the major sites of cancer and to leukaemia. The sites which have been selected for study with their respective International List (Detailed) numbers are :

| | | | |
|---|---|---|---|
| Buccal cavity | 140–148 | Urinary organs | 180–181 |
| Stomach | 151 | Breast (female) | 170 |
| Intestines | 152–153 | Uterus | 171–174 |
| Rectum | 154 | Genital organs (male) | 177–179 |
| Respiratory system | 161–163 | Leukaemia | 204 |
| | | All sites | 140–205 |

The period under review covers the years 1941 to 1958 inclusive. The year 1941 was selected as a suitable starting point because in that year, such sites of cancer as intestine and stomach began to be listed separately in the annual Vital Statistics reports for Canada.

The statistical procedures which have been used in the analysis of the data may be explained briefly as follows. First, the mortality rates for each site and sex were age-adjusted to the Canada 1951 census population as a standard. This procedure accounts for changes in rates which are due to changes in the age-structure of a population in the course of time. Since cancer death rates increase greatly with age and since the population of Canada is aging—that is, the relative number of older individuals is increasing, it is essential that this age-adjustment be carried out whenever any comparison of rates in time is made.

Second, the age-adjusted rates for the period 1941–1949 were made comparable with the years 1950–1958 by means of comparability ratios calculated by the Dominion Bureau of Statistics on the deaths in Canada in 1949. In that year the sixth revision of the International Classification of Diseases, Injuries and Causes of Death was published and, in order to assess the variations between this revision and the previous one, all deaths in Canada in 1949 were tabulated by both. From this dual tabulation the comparability ratios were calculated.

Third, the trend line for each set of age-adjusted rates was found. This line is, among all possible lines, the one for which the sum of the squared vertical distances from it to the points representing the rates is a minimum. It is commonly known as the " best-fitting " line. If this line tends to rise through the time interval under consideration (1941–1958) it has a positive slope. If the line falls the slope is negative and if it is horizontal the slope is zero. When the line has a positive or negative slope it signifies that the rates have increased or decreased during the interval and the probability is computed that a true rise or drop has occurred. This probability depends upon two factors : the magnitude of the slope of the line, and the amount of scatter of the actual points about the line. The probability value of less than 0·05 was considered sufficiently small to call a slope significantly different from zero. Hence, a "significant" trend indicates that the probability of obtaining, through chance alone, a set of rates which would give rise to this trend, or one at least as pronounced, is less than 5 in 100. It will be noted that the rates for some sites are increasing or decreasing but the subsequent tests of significance show that the increase or decrease may be due to chance variation. This illustrates the weakness of interpreting mortality trends from an inspection of death rates and points out the necessity for testing for significance.

The following example of the fitting of the trend line is based upon the age-adjusted death rates for cancer of the stomach in males.

*Example :* based upon age-adjusted death rates for cancer of the stomach in males.

| Year | Death rate ($Y$) | Time ($X$) | $Y'$ | $(Y - Y')$ |
|---|---|---|---|---|
| 1941 | 31·7 | −8·5 | 31·4 | 0·3 |
| 1942 | 30·9 | −7·5 | 31·0 | −0·1 |
| 1943 | 30·9 | −6·5 | 30·5 | 0·4 |
| 1944 | 29·9 | −5·5 | 30·1 | −0·2 |
| 1945 | 30·2 | −4·5 | 29·6 | 0·6 |
| 1946 | 28·2 | −3·5 | 29·2 | −1·0 |
| 1947 | 29·0 | −2·5 | 28·8 | 0·2 |
| 1948 | 27·9 | −1·5 | 28·3 | −0·4 |
| 1949 | 27·5 | −0·5 | 27·9 | −0·4 |
| 1950 | 26·6 | 0·5 | 27·4 | −0·8 |
| 1951 | 27·2 | 1·5 | 27·0 | 0·2 |
| 1952 | 28·0 | 2·5 | 26·6 | 1·4 |
| 1953 | 25·4 | 3·5 | 26·1 | −0·7 |
| 1954 | 25·6 | 4·5 | 25·7 | −0·1 |
| 1955 | 25·7 | 5·5 | 25·3 | 0·4 |
| 1956 | 25·0 | 6·5 | 24·8 | 0·2 |
| 1957 | 24·3 | 7·5 | 24·4 | −0·1 |
| 1958 | 23·9 | 8·5 | 24·0 | −0·1 |

$\Sigma Y$ = sum of $Y$ = 497·9.

$\bar{Y}$ = mean of $Y$ = 27·66.

$\Sigma XY$ = sum of products of $X$ and $Y$ = − 211·8.

$\Sigma X^2$ = sum of squares of $X$ = 484·5.

$$\text{SLOPE } (m) = \frac{\Sigma XY}{\Sigma X^2} = -\ 0{\cdot}437$$

$$Y' = mX + \bar{Y}$$

$$\Sigma(Y - Y')^2 = 5{\cdot}38$$

$$S_m = \text{standard deviation of slope} = \sqrt{\frac{\Sigma(Y - Y')^2}{(n-2)\ (\Sigma X^2)}} = \sqrt{\frac{5{\cdot}38}{16 \times 484{\cdot}5}} = 0{\cdot}026$$

$$t = \text{Student's ``t'' test} = \frac{|\,m\,|}{S_m} = \frac{0{\cdot}437}{0{\cdot}026} = 16{\cdot}8$$

The number of degrees of freedom is 16 when $n = 18$ and from the Table of "$t$" the probability that this trend is due to chance is less than 0·001 or one in one thousand.

It is concluded, therefore, that there has been a significant drop in the age-adjusted death rate for cancer of the stomach in Canadian males during this interval of time.

*Analyses of specific sites of cancer*

The age-adjusted mortality rates for the nine sites of cancer, for leukaemia, and for all sites combined are shown for males in Table I and for females in Table II. The trend line has been fitted to each set of rates and the appropriate test of significance made. The results may be summarized as follows :

TABLE I.—*Age-adjusted Mortality Rates Per* 100,000 *Population in Canada for Various Sites of Cancer—Male*

| Year | Buccal cavity | Stomach | Intestines | Rectum | Respiratory system | Urinary organs | Genital system | Leukaemia | All sites |
|---|---|---|---|---|---|---|---|---|---|
| 1941 | 7·2 | 31·7 | 15·0 | 7·1 | 9·1 | 7·4 | 12·9 | 3·8 | 123·2 |
| 1942 | 7·0 | 30·9 | 15·2 | 7·4 | 8·9 | 7·6 | 12·4 | 3·6 | 121·9 |
| 1943 | 7·1 | 30·9 | 15·6 | 7·5 | 9·9 | 7·8 | 12·6 | 3·9 | 124·7 |
| 1944 | 6·5 | 29·9 | 14·8 | 8·5 | 9·8 | 7·7 | 12·1 | 4·2 | 122·7 |
| 1945 | 6·1 | 30·2 | 15·6 | 7·8 | 10·4 | 7·7 | 11·9 | 4·0 | 123·5 |
| 1946 | 5·7 | 28·2 | 14·9 | 7·5 | 11·9 | 8·0 | 12·5 | 4·3 | 122·9 |
| 1947 | 5·9 | 29·0 | 15·2 | 7·9 | 13·3 | 8·1 | 13·5 | 4·8 | 128·9 |
| 1948 | 6·1 | 27·9 | 14·9 | 8·3 | 13·9 | 8·5 | 13·3 | 4·7 | 129·7 |
| 1949 | 5·9 | 27·5 | 14·9 | 7·8 | 15·6 | 8·1 | 14·1 | 4·9 | 131·0 |
| 1950 | 5·4 | 26·6 | 14·6 | 8·2 | 16·5 | 8·4 | 14·1 | 4·8 | 128·5 |
| 1951 | 4·9 | 27·2 | 14·0 | 7·0 | 17·3 | 8·4 | 13·4 | 5·2 | 131·4 |
| 1952 | 4·9 | 28·0 | 16·0 | 7·2 | 19·2 | 8·2 | 14·4 | 5·3 | 136·8 |
| 1953 | 4·7 | 25·4 | 14·9 | 8·0 | 20·4 | 8·3 | 14·5 | 6·3 | 136·4 |
| 1954 | 4·8 | 25·6 | 14·3 | 8·4 | 21·6 | 8·8 | 13·9 | 6·2 | 137·6 |
| 1955 | 5·2 | 25·7 | 14·5 | 8·8 | 22·9 | 9·4 | 14·1 | 6·1 | 141·0 |
| 1956 | 5·2 | 25·0 | 14·9 | 8·2 | 24·7 | 9·8 | 14·5 | 6·3 | 142·4 |
| 1957 | 4·7 | 24·3 | 15·2 | 8·4 | 25·4 | 9·1 | 13·8 | 6·4 | 142·5 |
| 1958 | 4·3 | 24·9 | 15·1 | 8·1 | 26·3 | 8·8 | 14·1 | 6·8 | 143·4 |

TABLE II.—*Age-adjusted Mortality Rates Per* 100,000 *Population in Canada for Various Sites of Cancer—Female*

| Year | Buccal cavity | Stomach | Intestines | Rectum | Respir-atory system | Urinary organs | Breast | Cervix uteri | Corpus uteri | Leu-kaemia | All sites |
|---|---|---|---|---|---|---|---|---|---|---|---|
| 1941 | 1·4 | 18·1 | 17·8 | 5·1 | 3·2 | 3·9 | 22·7 | 7·9 | 9·7 | 2·9 | 124·6 |
| 1942 | 1·7 | 17·9 | 18·1 | 5·1 | 3·1 | 4·2 | 22·5 | 7·1 | 11·3 | 3·0 | 124·6 |
| 1943 | 1·6 | 17·9 | 18·3 | 5·4 | 3·6 | 4·0 | 23·4 | 6·5 | 10·8 | 2·6 | 125·4 |
| 1944 | 1·4 | 16·8 | 19·8 | 5·3 | 3·9 | 4·3 | 22·1 | 7·0 | 10·4 | 3·4 | 124·9 |
| 1945 | 1·3 | 15·3 | 19·0 | 5·1 | 3·4 | 4·1 | 22·7 | 8·0 | 9·9 | 3·0 | 122·8 |
| 1946 | 1·2 | 16·8 | 18·9 | 5·2 | 3·4 | 3·9 | 23·3 | 6·9 | 10·2 | 3·5 | 123·8 |
| 1947 | 1·3 | 14·9 | 18·4 | 5·5 | 4·8 | 4·3 | 22·4 | 7·8 | 9·6 | 3·3 | 124·0 |
| 1948 | 1·3 | 16·1 | 18·7 | 5·5 | 3·7 | 4·1 | 23·5 | 7·6 | 9·0 | 3·4 | 126·1 |
| 1949 | 1·3 | 14·6 | 17·2 | 5·1 | 4·4 | 4·4 | 22·7 | 7·9 | 8·3 | 4·3 | 122·9 |
| 1950 | 1·2 | 14·3 | 18·0 | 4·8 | 3·3 | 4·6 | 22·9 | 8·0 | 7·3 | 4·5 | 121·0 |
| 1951 | 1·3 | 13·8 | 18·5 | 5·6 | 3·7 | 4·1 | 22·9 | 9·0 | 7·4 | 3·9 | 123·3 |
| 1952 | 1·3 | 14·0 | 17·8 | 5·2 | 4·0 | 4·0 | 23·2 | 8·7 | 6·7 | 3·9 | 121·0 |
| 1953 | 1·4 | 14·1 | 17·3 | 5·0 | 3·6 | 3·9 | 24·1 | 8·1 | 5·9 | 4·1 | 122·0 |
| 1954 | 1·4 | 13·6 | 16·8 | 4·8 | 3·8 | 4·0 | 23·2 | 7·9 | 6·8 | 3·5 | 120·9 |
| 1955 | 1·3 | 12·6 | 17·4 | 4·6 | 4·6 | 4·2 | 22·6 | 7·8 | 5·4 | 4·7 | 119·6 |
| 1956 | 1·4 | 12·6 | 16·6 | 5·1 | 4·1 | 3·9 | 22·8 | 8·3 | 5·7 | 4·3 | 119·6 |
| 1957 | 1·3 | 11·6 | 16·4 | 5·1 | 3·9 | 3·9 | 23·6 | 8·0 | 5·5 | 4·5 | 120·2 |
| 1958 | 1·2 | 11·4 | 17·0 | 5·1 | 3·8 | 4·0 | 23·2 | 7·6 | 5·6 | 4·7 | 118·5 |

*Buccal cavity.*—This analysis includes deaths assigned to cancer of the lip, tongue, salivary gland, floor of mouth, other parts of mouth, oral mesopharynx, nasopharynx, hypopharynx and pharynx (unspecified). The slopes of the trend lines show that there has been a significant decrease in mortality for males (38·6 per cent) but no change for females (Fig. 1).

Fig. 1.—Mortality trends in Canada—buccal cavity.
Male—significant. Female—not significant.

Fig. 2.—Mortality trends in Canada—stomach.
Male—significant. Female—significant.

*Stomach.*—The slopes of the trend lines for mortality from cancer of the stomach reflect significant declines for males and for females. Over the period 1941–1958 the male death rate has dropped 23·6 per cent and the female 35·5 per cent (Fig. 2).

*Intestines.*—It will be noted that the age-adjusted mortality rates for females are consistently higher than those for males throughout the period. The slope of the trend line for males shows no significant change but that for females shows a significant decrease (11·6 per cent) in the age-adjusted rates (Fig. 3).

*Rectum.*—The analysis of the deaths ascribed to cancer of the rectum indicates no significant change in either male or female mortality (Fig. 4).

*Respiratory system.*—This analysis includes deaths attributed to cancer of the trachea, bronchus and lung not specified as secondary. There has been a significant increase in male mortality but in females, although the trend line indicates an increase of 20 per cent between 1941 and 1958, the probability that this might be due to chance is about 0·07. In males the age-adjusted death rate has increased

Fig. 3.—Mortality trends in Canada—intestine. Male—not significant. Female—significant.

Fig. 4.—Mortality trends in Canada—rectum. Male—not significant. Female—Not significant.

271·4 per cent over the years 1941–1958 according to the fitted trend line, but as the graph indicates a single straight line does not produce a satisfactory fit throughout the whole period, the rate of increase being apparently greater after 1945 than it was during 1941–1945. (Fig. 5.)

*Urinary organs.*—The slopes of the trend lines indicate no significant change in the female rates. The male rate increased 24·3 per cent (Fig. 6).

FIG. 5.—Mortality trends in Canada—respiratory system.
Male—significant. Female—not significant.

FIG. 6.—Mortality trends in Canada—urinary organs.
Male—significant. Female—not significant.

FIG. 7.—Mortality trends in Canada—breast.
Female—not significant.

*Breast.*—The analysis of the deaths attributed to breast cancer in females indicates that no significant change in mortality occurred in the period 1941–1958 (Fig. 7).

*Uterus.*—Age-adjusted mortality rates have been calculated separately for deaths assigned to cancer of the cervix uteri and those assigned to the corpus

FIG. 8.—Mortality trends in Canada—uterus.
Uterus—significant.
Corpus—significant.
Cervix—significant.

FIG. 9.—Mortality trends in Canada—male genital.
Male—significant.

uteri. The trend line for cancer of the cervix shows an increase in the rates of 13·7 per cent which is of borderline significance ; the probability that the trend is due to chance lies between the 0·05 and the 0·02 levels of confidence. The slope of the trend line for the corpus uteri indicates a significant decrease (55·4 per cent) in the mortality rates. The slope of the trend line for the uterus including the cervix indicates a significant decrease of 28·7 per cent in the age-adjusted rates (Fig. 8).

*Male genital organs.*—This analysis includes deaths attributed to cancer of the prostate, testis and other male genital organs. The slope of the trend line indicates that a significant increase in mortality rates (16·9 per cent) occurred over the period being studied. (Fig. 9.)

FIG. 10.—Mortality trends in Canada—leukaemia.
Male—significant. Female—significant.

FIG. 11.—Mortality trends in Canada—all sites.
Male—significant. Female—significant.

*Leukaemia.*—The analysis shows a significant increase in the mortality rates for males and females. The male rates increased 91·4 per cent and the female rates 62·1 per cent (Fig. 10).

*All sites of cancer.*—The analysis of the deaths attributed to cancer of all sites, including leukaemia and the lymphatic and haemotopoietic system, shows that a significant increase has occurred in male mortality and a significant decrease in female mortality (Fig. 11).

### SUMMARY

An analysis has been made of the recorded deaths in Canada attributed to cancer during the period 1941–1958. The trends in the age-adjusted death rates for each site and sex have been tested for significant increases or decreases by means of Students' " *t* " test.

Significant decreases in mortality were found in

| | | |
|---|---|---|
| (*a*) | cancer of the buccal cavity . . . . . | male |
| (*b*) | cancer of the stomach . . . . . . | male |
| (*c*) | cancer of the stomach . . . . . . | female |
| (d) | cancer of the intestine . . . . . . | female |
| (e) | cancer of the corpus uteri . . . . . | |
| (f) | cancer of all sites . . . . . . . | female |

Significant increases in mortality were found in

| | | |
|---|---|---|
| (a) | cancer of the respiratory system . . . . | male |
| (b) | cancer of the urinary organs . . . . | male |
| (c) | cancer of the cervix uteri . . . . . | |
| (d) | cancer of the genital organs . . . . . | male |
| (e) | leukaemia . . . . . . . . . | male |
| (f) | leukaemia . . . . . . . . . | female |
| (g) | cancer of all sites . . . . . . . | male |

No significant changes in mortality were found in

| | | |
|---|---|---|
| (a) | cancer of the buccal cavity . . . . . | female |
| (b) | cancer of the intestine . . . . . . | male |
| (c) | cancer of the rectum . . . . . | male |
| (d) | cancer of the rectum . . . . . . | female |
| (e) | cancer of the respiratory system . . . . | female |
| (f) | cancer of the urinary organs . . . . | female |
| (g) | cancer of the breast . . . . . . | female |

The assistance of the Dominion Bureau of Statistics is gratefully acknowledged.